# Sulfur-Mediated Polycarbonate Polyurethane for Potential Application of Blood-Contacting Materials

**DOI:** 10.3389/fbioe.2022.874419

**Published:** 2022-03-09

**Authors:** Peichuang Li, Wanhao Cai, Xin Li, Hong Zhang, Yuancong Zhao, Jin Wang

**Affiliations:** ^1^ Heze Branch, Qilu University of Technology (Shandong Academy of Sciences), Biological Engineering Technology Innovation Center of Shandong Province, Heze, China; ^2^ Key Laboratory of Advanced Technologies of Materials, Ministry of Education, School of Materials Science and Engineering, Southwest Jiaotong University, Chengdu, China; ^3^ Institute of Physical Chemistry, Albert-Ludwigs-Universität Freiburg, Freiburg, Germany; ^4^ Department of Cardiology, Third People’s Hospital of Chengdu Affiliated to Southwest Jiaotong University, Chengdu, China

**Keywords:** sulfur, polycarbonate polyurethane, nitric oxide, biocompatibility, blood-contacting

## Abstract

In this study, a sulfur-mediated polycarbonate polyurethane (PCU-SS) is developed by mimicking the catalyzing ability of glutathione peroxidase (GPx) on nitric oxide (NO) in the human body. The PCU-SS is endowed with the capability to produce NO based on disulfide bonds, which could strongly improve the biocompatibility of the materials. The characterization results indicate that PCU-SS could not only decrease the adhesion of platelets but also enhance the capability of anti-thrombus. Moreover, it is shown that PCU-SS has a good compatibility with endothelial cells (ECs), while has a marked inhibition capacity of the proliferation of smooth muscle cells (SMCs) and macrophages (MA). Meanwhile, the result of animal implantation experiments further demonstrates the good abilities of PCU-SS on anti-inflammation, anti-thrombus, and anti-hyperplasia. Our results offer a novel strategy for the modification of blood-contacting materials based on disulfide bonds. It is expected that the PCU-SS could shed new light on biocompatibility improvement of cardiovascular stents.

## Introduction

Since the first performance of percutaneous coronary angioplasty in 1977 (Grüntzig, 1978), the direct intervention of interventional therapy has been widely used in improving the living quality of patients. The vascular interventional technique (VIT) is an important branch of interventional therapy (Cao et al., 2021; Pan et al., 2021), which is commonly used in the treatment of cardiovascular diseases (e.g., atherosclerosis). Although the clinical application is quite mature, adverse clinical events (Daemen et al., 2007; Faxon, 2012; Qiu et al., 2021) including late thrombosis and intimal hyperplasia are still long-term side effects of the VIT. To reduce the adverse events, the quality of interventional implants, especially the biocompatibility of interventional implants, is urgently required. The developed techniques can be roughly divided into several types. One important technique is tissue engineering including cell seeding (Zhu et al., 2008; Raina et al., 2014), which could be applied to vascular stent surfaces to achieve surface endothelialization. Although this method can achieve the closest state of the normal tissue, the endothelial tissue is restricted by cumbersome procedures and the significant risk of tissue shedding during implantation. As another choice, bulk modification and surface treatment are more popular in clinical studies (Bekmurzayeva et al., 2018; Oliver et al., 2021) since the process of material structure modifications is maneuverable and the stability of modified implants is robust.

The modification of the structure and function of materials can be optimized by means of physical blending or chemical synthesis. Physical means such as the drug loading (Roopmani et al., 2019; Wang et al., 2021) or functional component doping (Xu et al., 2019) can significantly improve the biocompatibility of vascular interventional implants. However, overuse of anticoagulants and non-pharmaceutical component doping may lead to a high risk of thrombocytopenia and hemorrhage. As a comparison, the chemical means seem more promising since multiple biological functions and safety can both be achieved (Chen et al., 2016; Xu et al., 2018). For instance, the introduction of polymer brushes for surface modification (Yang et al., 2014) could reduce the adhesion of blood components to obtain a long-term anticoagulation effect. The introduction of zwitterionic components (Chen et al., 2016; Lv et al., 2021) can further effectively prevent the attack of blood to build an anti-fouling surface.

To date, the chemical coating of the stent surface has been extensively used in the drug-eluting stent (DES) implantation, which remains the primary modality for patients with acute atherosclerosis (Giri and Halaby, 2021; Saito and Kobayashi, 2021). Under the effect of drugs, various problems such as hyperplasia, thrombosis, and inflammation of atherosclerotic sites can be inhibited effectively in the early stage. Nevertheless, delayed healing of the endodermis and insufficient biocompatibility of the drug-carrying coating will bring great hidden danger to the restenosis and thrombosis in the late stage (Yang et al., 2015; Giri and Halaby, 2021). Therefore, it is necessary to design novel materials or modify existing polymers to solve the problems. Considering the existing modification methods, bulk modification can realize the change of the bulk property without worrying about the change caused by the loss of the interface quality (Li et al., 2020), whereas the surface modifications are flexible and changeable (Mani et al., 2007; Qiu et al., 2021; Yu et al., 2021). By combining the bulk modification and surface modification together, an optimizing strategy for the modification is expected.

**FIGURE 1:**
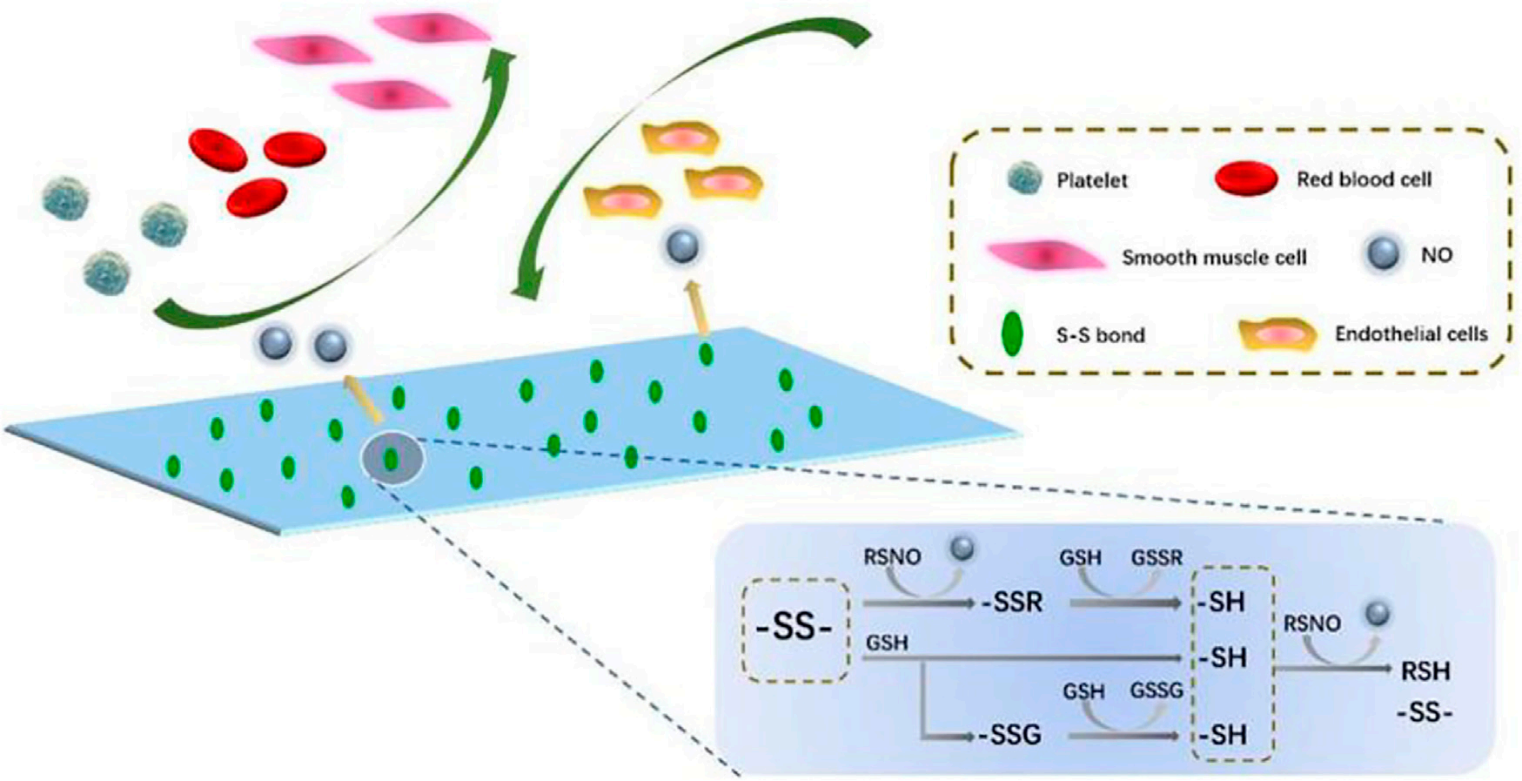
Diagram of the action mechanism of PCU-SS applied to the blood microenvironment. Disulfide bonds can catalyze the NO release from RSNO (generic term of endogenous NO donors) (Zhou et al., 2011). A part of NO is from the direct decomposition of disulfide bonds to the RSNO; the other comes from the exchange of S-S and–SH.

In this study, polyurethane (PU) as a popular polymer for the manufacture of advanced medical equipment is chosen as the fundamental polymer (Gostev et al., 2018; Naureen et al., 2021). Meanwhile, polycarbonate is found to have a great application potential in the surface modification of blood-contacting implants. Therefore, polycarbonate glycol is chosen as one of the raw materials of the final product (Wang et al., 2013; Ye et al., 2020). Then, PU is modified *via* disulfide bonds for improving the biocompatibility by mimicking the structural and functional characteristics of glutathione peroxidase (GPx) in the human body (Freedman et al., 1995; Wolin, 2011). GPx has the ability to catalyze the release of NO from endogenous NO donors (RSNO), which could not only prevent platelet adhesion and SMC proliferation but also combat inflammation and promote re-endothelialization (Freedman et al., 1995; Yang et al., 2018; Fan et al., 2019). In other words, the proposed PCU material (PCU-SS) is endowed with the capability to produce NO based on the formation and fracture of disulfide bonds (Cha and Meyerhoff, 2007; Zhou et al., 2011), as shown in Figure 1. A series of blood compatibility evaluations of PCU-SS show that platelet adhesion and the blood clot formation could be inhibited markedly. Cell cultures confirm that PCU-SS has a certain compatibility of endothelial cells (ECs), while it could also markedly inhibit the proliferation of smooth muscle cells (SMCs) and macrophages (MA). Animal experimental results further show that the histocompatibility and the ability to inhibit thrombus formation and intimal hyperplasia of PCU-SS are much higher than that of the unmodified samples. Overall, our strategy based on disulfide bonds opens up a new avenue for the preparation of new blood-contacting materials.

## Materials and Methods

### Materials and Reagents

Diphenylmethane-4,4′-diisocyanate (MDI), stannous octoate, S-nitrosoglutathione (GSNO), and l-glutathione (GSH) are all obtained from Sigma-Aldrich. Polycarbonatediol (PCDL) is purchased from UBE Industries, Ltd. 2-Hydroxyethyl disulfide (BHS) is purchased from TCI. 1,4-Butylene glycol (BDO) and N, N-Dimethylacetamide (DMAc) are obtained from Kelong Chemical Reagent Co., Ltd (Chengdu, China) and distilled under vacuum before use.

### Preparations of PCU-SS and PCU-BDO

**FIGURE 2:**
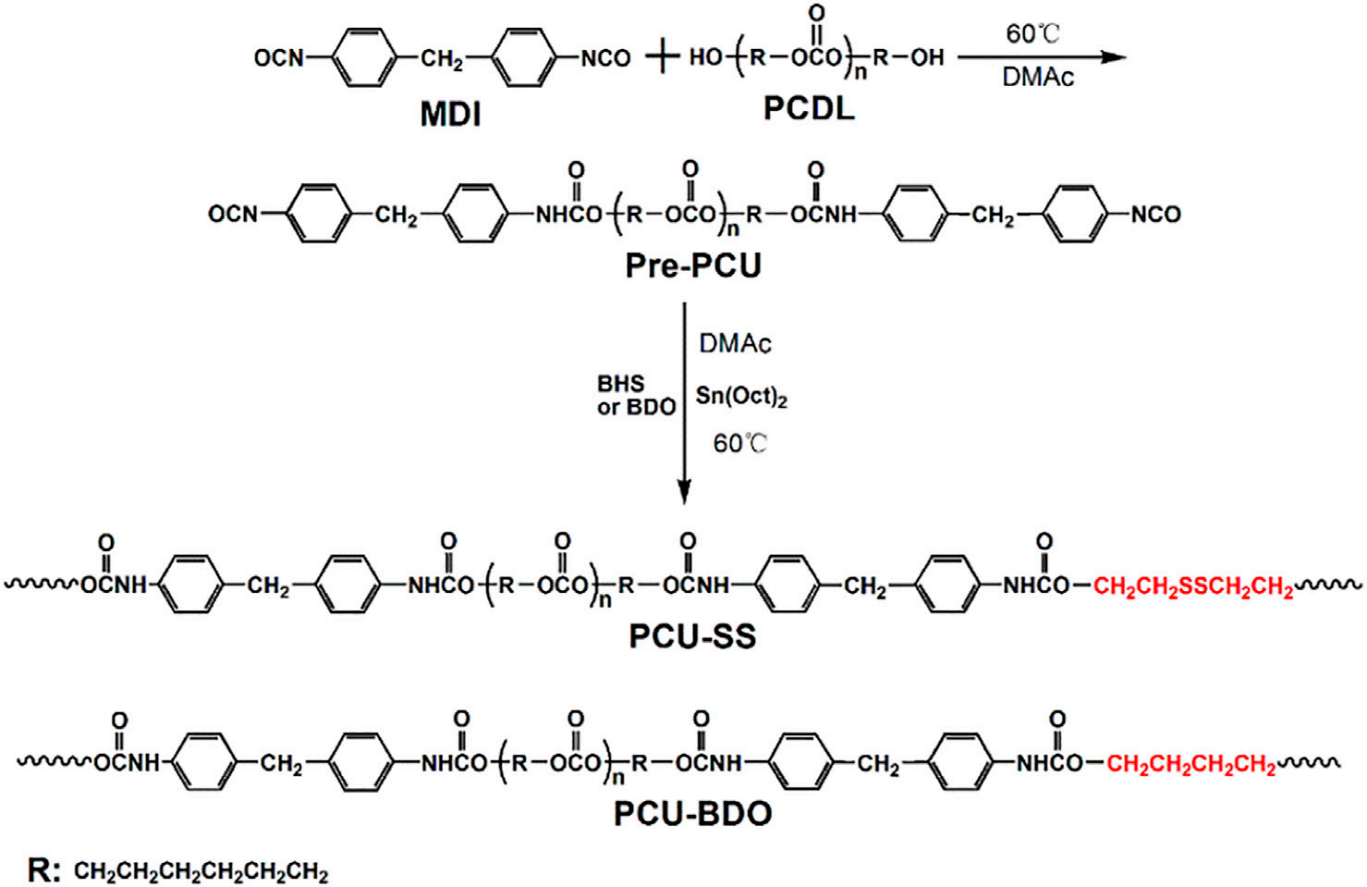
Synthetic route of PCU-SS and PCU-BDO.

According to our previous studies (Li et al., 2020), PCU-SS is synthesized by stepwise polymerization. In particular, PCU-BDO without disulfide bonds is selected as the control sample. The synthesis can be divided into two steps (Figure 2): first, PCDL is added into a flask and dried at 80°C under high vacuum for 4 h and then cooled down for standby application. Then, moderate PCDL is dissolved in DMAc and poured into a three-neck flask. Subsequently, MDI is added into the reaction flask under an argon atmosphere. After that, the aforementioned reaction mixture is allowed to react at 60°C for 2 h. Then, BHS or BDO, as the chain extender, and stannous octoate (0.1%), as the catalyst, are added into the flask successively. Subsequently, the aforementioned mixture is allowed to react at 60°C for 6 h. The feed ratio of the MDI/PCDL/chain extender is 2/1/1. On the predetermined time, the final reaction solution is first cooled to room temperature and then added into methanol to precipitate the final product. The resulting polymer is finally thoroughly washed with ethanol and distilled water successively and then transferred to a vacuum drying oven for further use.

### Characterization of PCU-SS

In order to verify the structural information of PCU-SS, Fourier transform infrared spectroscopy (FTIR, Nicolet 5,700) is used to identify the major functional groups of PCU-SS, while the proton nuclear magnetic resonance (^1^H NMR) using a Bruker AV II-400 spectrometer is further used for detailed structural analysis.

### Preparation of PCU-SS Coating

For the procedure, 316L stainless steel (316L) sheets (diameter = 10 mm and thickness = 1 mm), after polishing, cleaning, and drying, are placed in a polytetrafluoroethylene mold. The moderate polymer is dissolved in tetrahydrofuran (THF) to prepare the 10% polymer solution. The obtained solution is then added into the PTFE mold, and 316L sheets are immersed in the solution. After the evaporation of the solvent, PCU-SS coating could be prepared on the surface of 316L sheets.

### Catalytic Release of NO

The NO catalytic release behavior of the PCU-SS sample is measured by the chemiluminescence method (Coneski and Schoenfisch, 2012; Fan et al., 2019) using real-time NO analysis equipment (NOA 280i). Before the measurement, the operating temperature and pressure of equipment are adjusted to the working state. Then, the phosphate-buffered saline (PBS, pH = 7.4) solution containing 10 μM GSNO and 10 μM GSH is added into the reaction vessel, which is then filled by high-purity nitrogen gas stream. Subsequently, the test sample is immersed into the solution until the signal collected by the computer is stable and completely captured.

### Blood Compatibility

#### Platelet Adhesion

The platelet-rich plasma (PRP) is prepared in advance *via* centrifuging fresh human whole blood at l500 rpm for 15 min. In particular, this experiment is approved by the Local Ethical Committee and Human Subject Protocol of China, following the ethical rules. Before the test, the samples are divided into two groups including donor groups (+GSNO) and non-donor groups (-GSNO). Then, the samples are placed into a 24-well plate, and then, the equal volume (80 μl) of PRP is added onto the sample surface. After that, the plate containing samples is transferred into an incubator. As the NO donors, GSNO (10 μM) and GSH (10 μM) are specially added into the PRP. After 1 h incubation at 37°C, all the samples are rinsed with PBS to remove the excess PRP and non-firmly adsorbent platelets from the sample surface. Immediately, the samples are immersed into a glutaraldehyde fixative (2.5 wt%). After fixing overnight, all the samples are rinsed again with PBS. Subsequently, the samples are then dehydrated, dehydrated (query), dried, and sputtered with gold. Finally, scanning electron microscopy (SEM) is employed to observe the morphology and quantity of adherent platelets of different samples.

#### Dynamic Evaluation of Hemocompatibility

All animal experiments are approved by the Ethics and Welfare Committee of Southwest Jiaotong University, and they meet the requirements of Laboratory Animal Administration Rules. The dynamic evaluation of hemocompatibility (Fan et al., 2019; Qiu et al., 2021; Yu et al., 2021) is carried out using adult New Zealand white rabbits with a weight of 3.5–4.0 kg. First, 316L foils (8 × 10 mm) are respectively coated with PCU-BDO and PCU-SS by the solvent evaporation method. Then, the samples are curled and then inserted into the heparinized sterile catheters. After the isolation of the right jugular vein and left carotid artery, surgical indwelling needles are connected with the carotid artery and jugular vein, respectively. Immediately, sterile catheters containing samples are connected with the indwelling needles to build a blood circulation model. Simultaneously, 10 mΜ GSNO and 10 mΜ GSH are injected into the blood system through the ear vein. Dynamic evaluation is stopped after 1 h, and the cross section close to the sample and the clot on the foil surface are recorded. Then, the thrombus harvested on the foil surface is immersed into the solution of glutaraldehyde (2.5 wt%) and then respectively dehydrated, dealcoholized, and weighed. Finally, the microtopography of different samples is observed by SEM.

### Cytocompatibility Evaluation

#### EC Culture

Before culture, the experimental samples are divided into donor groups and non-donor groups. The culture medium consisted of 85% M199 medium (Hyclone) and 15% fetal bovine serum (FBS), and the culture medium of donor groups is supplemented with NO donors (10 μM GSNO and 10 μM GSH). EC are seeded on the samples with a density of 1.5 × 10^4^ cells/sample and incubated for 4 h, 1 day, and 3 days in a standard cell incubator. NO donors are replenished in the culture process every 24 h. Besides, the EC viability is analyzed *via* the cell counting kit-8 (CCK-8, Dojindo) assay. Before each culture node, 4 μg/ml of calcein-AM (Cal-AM) is mixed with the medium, and then, the cells on the sample surface can be stained. Subsequently, all the samples are cleaned and fixed successively and then observed using a fluorescence microscope (IX51, Olympus).

#### SMC Culture

Different from EC culture, the SMC culture medium consisted of the Dulbecco’s modified Eagle medium/F12 (Hyclone) and FBS (15%). The seeding density of SMC is 2.0 × 10^4^ cells/sample, and the culture medium of donor groups is also supplemented with NO donors. Then, all the samples are incubated in a standard incubator (37°C, 5% CO_2_). At the scheduled time (4 h, 1 day, and 3 days), SMC is stained using Cal-AM and then observed *via* a fluorescence microscope. Moreover, SMC viabilities at 1 day and 3 days are measured using CCK-8.

#### MA Culture

In this study, mouse mononuclear macrophages (RAW 246.7) are used for the analysis of the MA growth behavior. MA is cultured in a high Gly medium (Hyclone) supplemented with 15% FBS and then seeded on the samples with a density of 5.0 × 10^4^ cells/sample. NO donors are used as control variables to investigate changes in the macrophage growth. After being respectively cultured for 4 h, 1 day, and 3 days, the MA is stained by Cal-AM, fixed with 2.5% glutaraldehyde, and observed using a fluorescence microscope. In addition, the cell culture medium of the MA cultured for 3 days is also collected. The interleukin-6 (IL-6) and tumor necrosis factor (TNF-α) contents in the medium are detected by using the enzyme-linked immunosorbent assay (ELISA) kit to evaluate the anti-inflammatory ability of different samples.

### Subcutaneous Implantation

In this study, the dorsal skin of Sprague–Dawley (SD) rats is used for short-term (3 weeks) and long-term (9 weeks) subcutaneous embedding (Li et al., 2020). At the predetermined time, fibrous capsules formed around the samples are collected and then soaked in paraformaldehyde at ambient temperature for 72 h. Then, the fixed samples are processed through a series of procedures including cleaning with PBS, fixing with paraformaldehyde, dehydrating with graded ethanol, saturating with xylene, and embedding with paraffin. Subsequently, the paraffin samples are placed on a rotary slicer for sectioning. After that, hematoxylin and eosin (HE) are used to stain the obtained paraffin sections. At last, stained slices are observed and then photographed with a microscope.

### Aortic Implantation

Before implantation, PCU-SS coating is prepared on the surface of 316L wires using the ultrasonic atomizing spray. Adult SD rats (male, *n* = 12) are used in the aortic implantation. First, the rats are injected with pentobarbital sodium for anesthesia. Then, the abdominal aortas of rats are carefully isolated. Next, the coated wires are implanted into the aortas. After vascular suture, the abdominal cavities of rats are treated with gentamicin for anti-inflammation. Besides, the rats are injected with penicillin, and the skin suture sites are treated with iodophor for postoperative anti-inflammatory therapy. After 30 days, the implanted samples together with the blood vessels are collected and then fixed with paraformaldehyde. Subsequently, the fixed samples are photographed *via* SEM and stained *via* the immunofluorescence technique after several processes (dehydration, dealcoholization, and drying).

### Stent Implantation

Adult New Zealand white rabbits (male, *n* = 3) as model animals are used for stent implantation experiments. PCU-SS coating is prepared on the 316L stents using the ultrasonic atomizing spray. First, bare stents and coated stents together with balloons are compressed *via* a pressure holding device, and the rabbits are anesthetized with pentobarbital sodium. Then, the iliac arteries are isolated, and heparin is used for systemic anticoagulant treatment in rabbits. Immediately, the stents are inserted into the iliac arteries on both sides through percutaneous puncture. After the balloon is pulled out from the iliac artery, the blood vessels and tissues are sutured quickly. In the first 3 days after stent implantation, all rabbits are injected with penicillin and fed with warfarin sodium daily for postoperative anti-inflammatory and anticoagulant treatment. Subsequently, the postoperative rabbits are routinely fed for 27 days. After that, the stents attached to the vessels are collected and then soaked in paraformaldehyde. Finally, the lumens of the stents are observed by SEM, and then, the cross sections of the stents are photographed *via* a microscope after hard tissue embedding.

### Statistical Analysis Method

All data are presented as mean ± standard deviation (SD). Statistical analysis is assessed by analysis of variance (ANOVA). Statistical significance is set at the value of *p* < 0.05, 0.01 and 0.001.

## Results and Discussion

### Structural Analysis of PCU-SS

**FIGURE 3:**
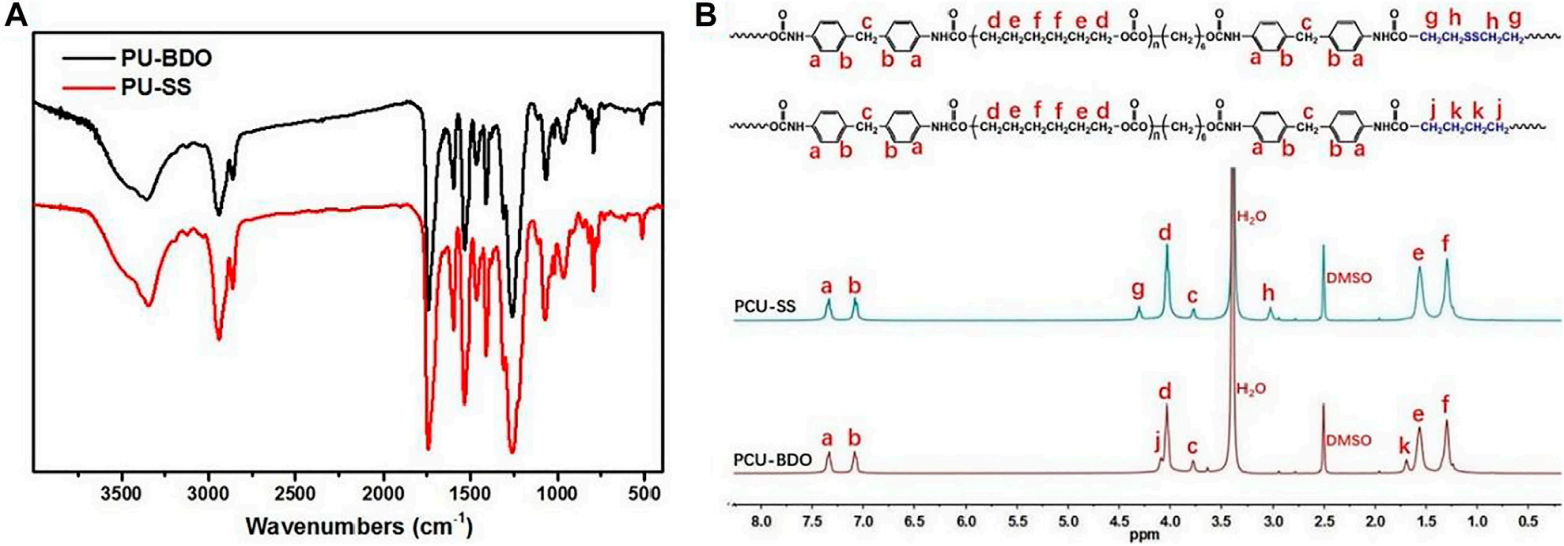
**(A)** FTIR spectra and **(B)** ^1^H NMR spectra of PCU-BDO and PCU-SS.

Different methods are used to characterize the synthesized PCU-SS. First, the main functional groups of PCU-SS are detected by FTIR (Figure 3A). The absorption peaks at 2,939 cm^−1^ and 2,859 cm^−1^ are attributed to the methylene group, while the peaks at 1741 cm^−1^ and 1,599 cm^−1^ are attributed to the carbonyl group and aromatic benzene ring, respectively. Furthermore, the peak belonging to the secondary amine of the carbamate group is observed at 1,532 cm^−1^, while the peak belonging to isocyanate does not appear in the range from 2,240 cm^−1^ to 2,280 cm^−1^. These results preliminarily proved the success of PCU-SS synthesis.

Detailed structural features are further detected by ^1^H NMR (Figure 3B). The chemical shift peaks at 4.03, 1.57, and 1.30 ppm are attributed to the methylene proton of the PCDL segment. The proton peaks at 7.33, 7.08, and 3.77 ppm are attributed to the MDI segment. Moreover, the proton peaks assigned to BDO and BHS exhibit different chemical shifts due to the chemical environment. The peaks at 4.30 ppm and 3.03 ppm belong to the BHS segment in PCU-SS, and the chemical shifts of 4.09 ppm and 1.69 ppm are attributed to the BDO segment in PCU-BDO. In general, ^1^H NMR analysis on the basis of FTIR further demonstrates the successful preparation of materials.

### *In vitro* NO Catalyzed Release and Platelet Adhesion of PCU-SS

**FIGURE 4:**
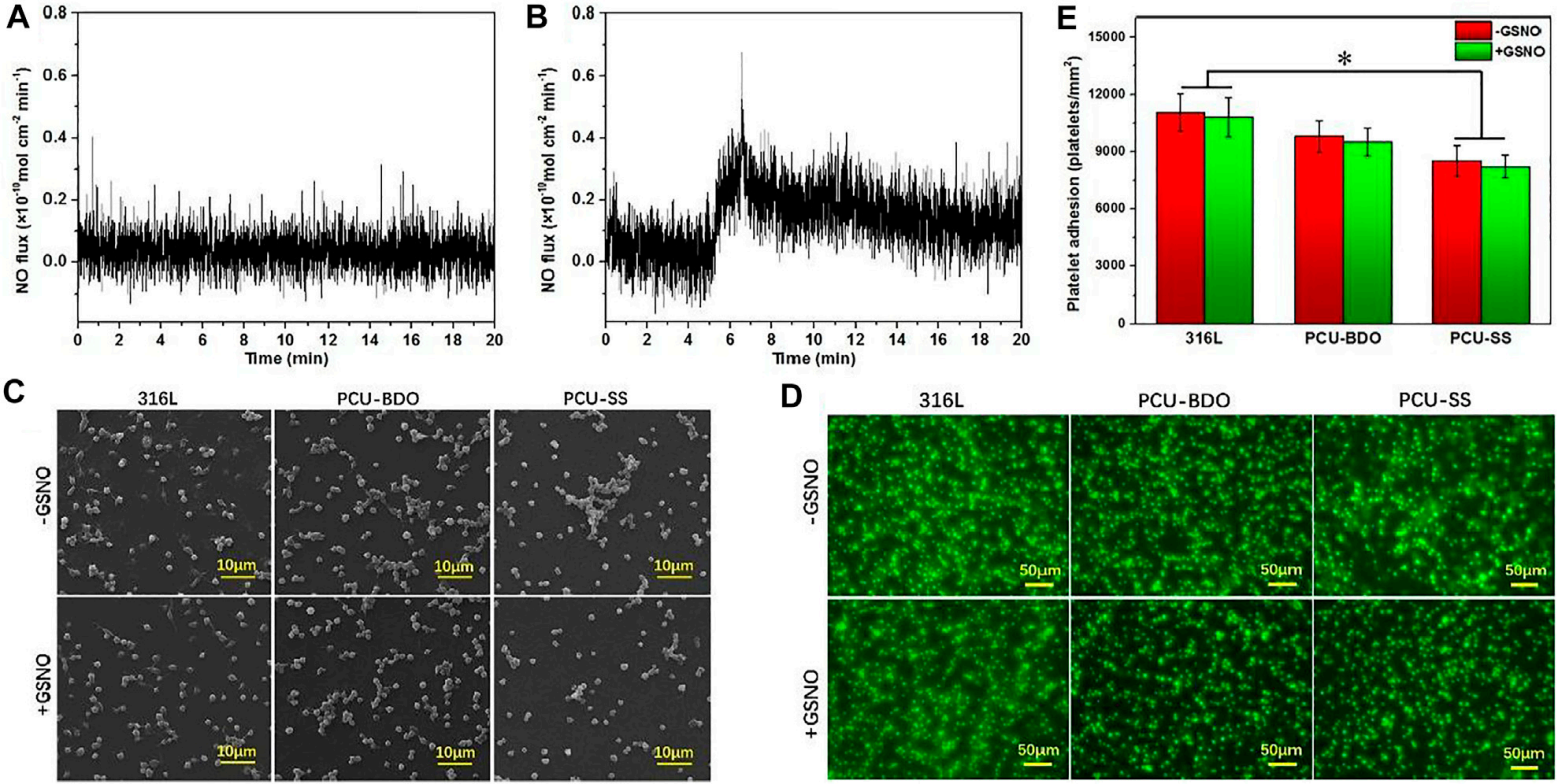
**(A)** Catalytic NO generation results of PCU-BDO and **(B)** PCU-SS in PBS containing the NO donor **(C)** SEM results **(D)** fluorescence staining, and **(E)** count results of platelet adhesion on the surface of 316L, PCU-BDO, and PCU-SS (*p < 0.05, mean ± SD, *n* = 5).

As an important signal medium in the blood, NO plays a vital role in many physiological processes including maintaining vascular diastolic pressure and vascular homeostasis. Insufficient production of NO is associated with thrombosis and intimal hyperplasia (Bohl and West, 2000). Hence, the extra release of NO from implant device surfaces can yet be regarded as an effective strategy for the vascular implant apparatus. As described earlier, the endogenous NO donor could be decomposed and release NO in the presence of GPx. By mimicking the function and structure of GPx, the introduction of disulfide bonds in polyurethane can achieve the additional catalytic release of NO on the surface of medical devices. Here, the catalytic release of NO of both PCU-SS and PCU-BDO are evaluated *via* chemiluminescence (Figures 4A,B). It is found that the release curve of PCU-BDO is stable, and no apparent release is observed. In contrast, the NO release from PCU-SS exhibited a remarkable catalytic capacity (0.1–0.3 × 10^–10^ mol × cm^−2^ × min^−1^). The overall release rate of PCU-SS is close to that of normal endothelial cells. The significant release of NO mediated by disulfide bonds could offer promising effects for many biological behaviors of the material.

Platelets could adhere onto the sample surface *via* non-specific interactions such as the van der Waals forces and hydrogen bonds (Cai et al., 2019; Cai et al., 2022). Here, the platelet adhesion is evaluated by SEM and using a fluorescence microscope. From the SEM images (Figure 4C), it can be seen that platelet adhesion and activation are the most severe for 316L, while they are the lightest for PCU-SS. With the addition of GSNO, the catalyzing capacity of PCU-SS takes effect, which could further reduce the platelet adhesion. Similar results are also observed in the fluorescence microscope images where adherent platelets are stained with rhodamine (Figure 4D), as well as in the statistical results (Figure 4E). All these results indicate that PCU-SS with GSNO can effectively inhibit platelet adhesion, aggregation, and activation.

### Blood Circulation

**FIGURE 5:**
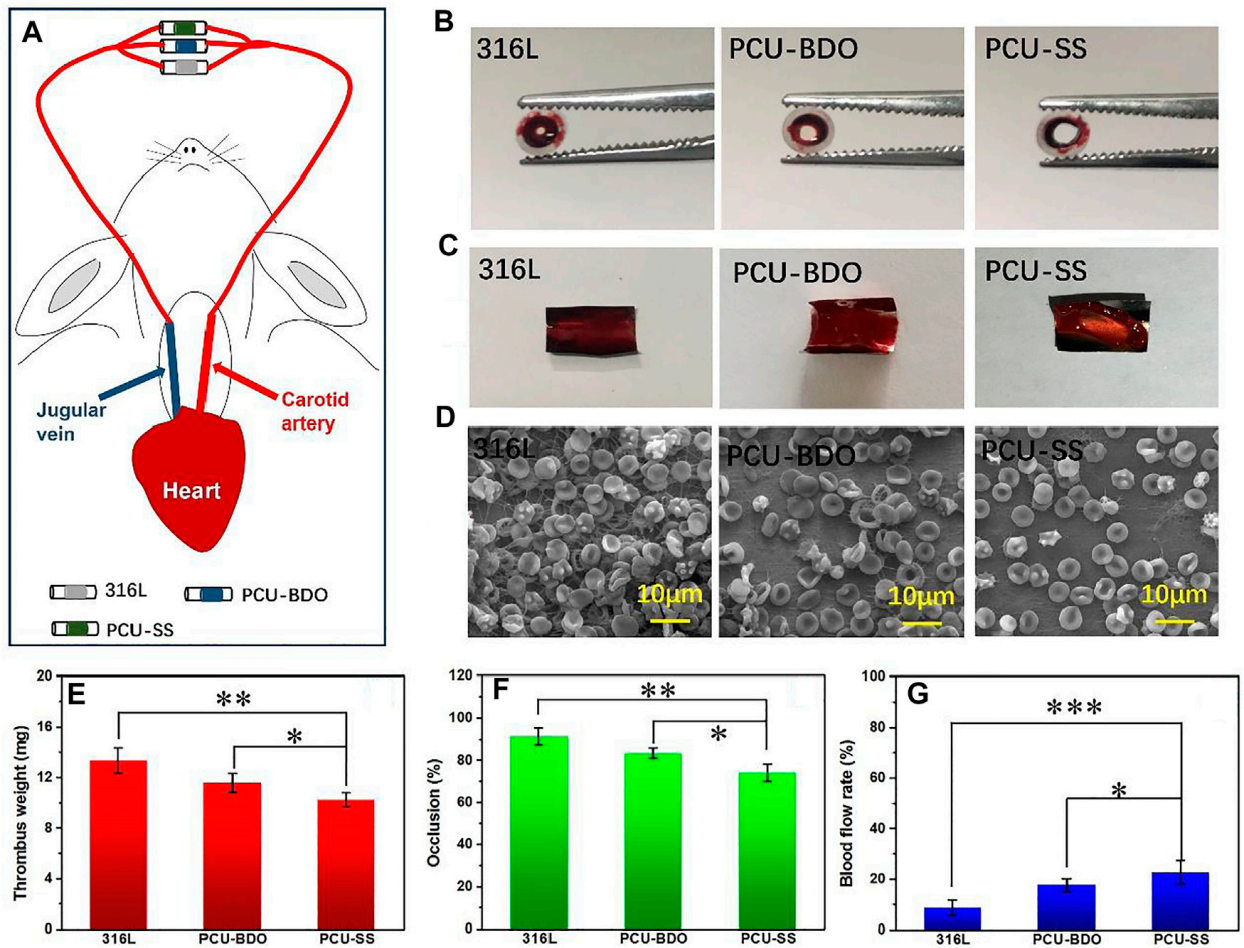
Blood circulation experiment **(A)** Schematic diagram of experiments **(B)** Digital photos of the catheter containing samples and **(C)** thrombi after circulation **(D)** SEM images of thrombi on the sample surface **(E)** Weighting results after dehydration **(F)** Statistical results of the occlusion rate and **(G)** blood flow rates of different samples (*p < 0.05, **p < 0.01, ***p < 0.001, mean ± SD, *n* = 5).

To evaluate the anticoagulant ability of PCU-SS, New Zealand white rabbits are used to build a blood circulation model (Figure 5A). After blood circulation, the catheters containing samples are removed, and the relevant treatment is proceeded. As shown in Figure 5B, the cross section of the catheter containing the 316L sample is almost completely blocked by thrombosis. The blockage of PCU-BDO is slightly improved, but serious thrombus still existed. In contrast, PCU-SS shows minimal blockage. Combined with the relevant statistical results in Figure 5F, the occlusion rate of PCU-SS is significantly lower than that of the other two groups. Compared with the initial stage of the experiment, the blood flow rates of 316L, PCU-BDO, and PCU-SS are 8.7, 17.6, and 22.7% of the initial speed (Figure 5G), respectively. These results show that PCU-SS can maintain normal blood circulation to the maximum extent.

Then, the samples taken from the catheters are photographed for the analysis of the thrombus. It can be seen from Figure 5C that the 316L sample is almost fully covered by thrombus, while the PCU-BDO and PCU-SS samples are also covered but partially by thrombus. Weight analysis (Figure 5E) shows that the thrombus weights of 316L and PCU-BDO are 13.3 mg and 11.6 mg, respectively, while the thrombus weight of PCU-SS is the lowest (10.2 mg). Further SEM results (Figure 5D) are used to illustrate the coagulation conditions of different sample surfaces. These macroscopic results are further validated by SEM: The surfaces of 316L and PCU-BDO are covered by a network structure including fibrinogen, red blood cells, and platelets. By contrary, the PCU-SS surface has the least blood components, indicating the best anticoagulant ability among the three.

### EC Compatibility

**FIGURE 6:**
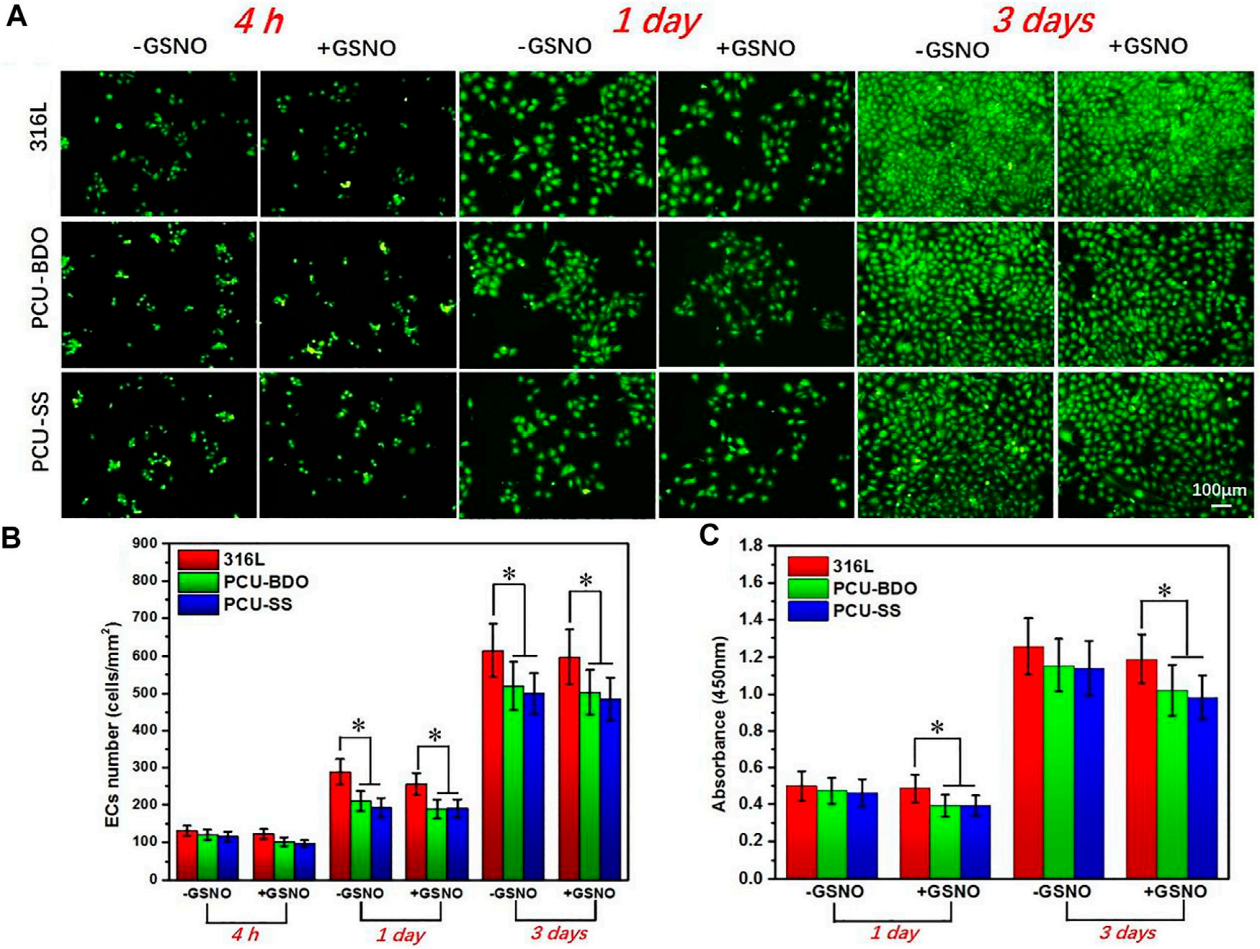
**(A)** Fluorescence results **(B)** cell count, and **(C)** CCK-8 results of EC (*p < 0.05, mean ± SD, *n* = 5).

Rapid endothelialization after implantation is beneficial to inhibit restenosis and prevent thrombosis; hence, it is an important index in evaluating the property of materials. Figure 6 shows the fluorescence results of adhesion and proliferation of EC *in vitro*. After 4 h, there is no marked difference between the three groups. A clear difference emerges when the culture time is extended to 1 day and 3 days. 316L showed the largest amount of EC adhesion, while the EC numbers on the surface of the PCU-BDO and PCU-SS are slightly lower than that of 316L. Further quantitative analysis (Figures 6B,C) of the adhesion number and cell activity of EC indicates that PCU-SS is not inferior to 316L for EC and hence can be considered to have good endothelial compatibility.

### SMC Culture

**FIGURE 7:**
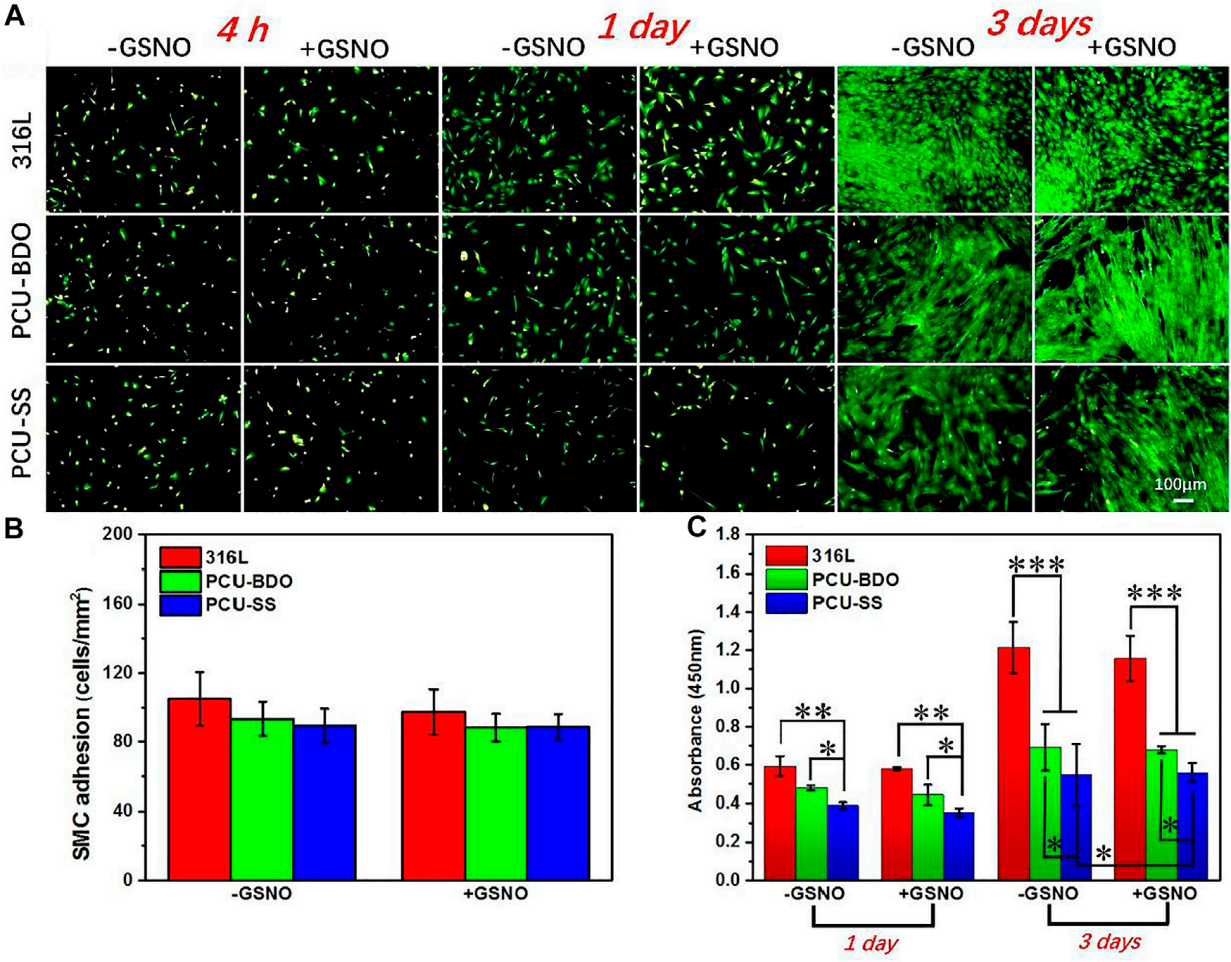
**(A)** Fluorescence staining **(B)** adhesion count of 4 h, and **(C)** CCK-8 detection of SMCs on the surface of different samples (*p < 0.05, **p < 0.01, ***p < 0.001, mean ± SD, *n* = 5).

SMC plays an important role in maintaining the elasticity of normal blood vessels. However, after implantation of interventional devices such as the intravascular stent, excessive proliferation of SMCs often leads to restenosis. Therefore, the anti-proliferation capacity of SMCs is also an important index in evaluating the property of materials. As shown in Figures 7A,B, at the first 4 h, there is no marked difference between the three samples since the cell morphology is not fully developed yet. The clear difference emerges when the culture time is extended. After 1 day, the SMC growth on the 316L surface is the best, while PCU-SS already shows the least adhesion number of SMCs. After 3 days, the coverage area of SMCs on the PCU-SS surface is the smallest, while the other two are almost fully covered by SMCs. The subsequent CCK-8 cell viability test (Figure 7C) also shows that PCU-SS is the most effective surface for inhibiting the proliferation of SMCs. In particular, the SMC vitality of PCU-SS at 3 days will further be suppressed after GSNO addition. This feature will have an effect on the inhibition of intimal hyperplasia after intravascular implantation.

### Subcutaneous Implantation

**FIGURE 8:**
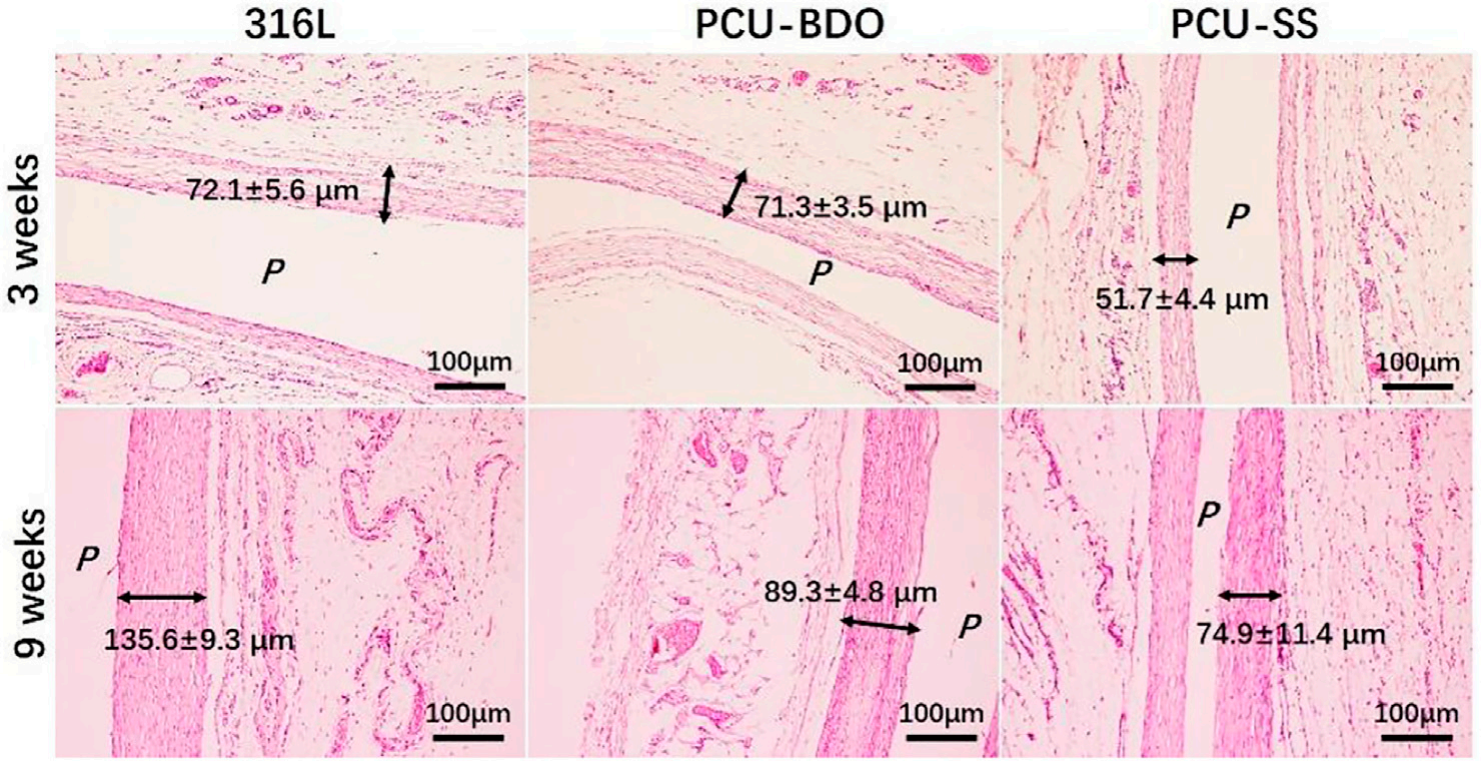
HE staining results of fibrous capsules formed around different samples (*P* is the implantation location).

Then, to test the anti-inflammation property, 316L sheets before and after coating are implanted subcutaneously in SD rats for 3 and 9 weeks, respectively. Fibrous capsules will be formed around different samples after implantation, and the HE staining results are shown in Figure 8. At 3 weeks of implantation, the thickness fibrosis around PCU-SS is the thinnest (51.7 ± 4.4 μm), while those around 316L and PCU-BDO are much thicker. At 9 weeks of implantation, all fibrosis become denser and thicker. It still can be seen that stimulation of the surrounding tissues by PCU-SS is minimal. Moreover, results of fluorescence staining (see Supplementary Figure S1 for details) and inflammatory factor release (see Supplementary Figure S2 for details) in MA culture *in vitro* also exhibit the inhibitory effect on inflammatory cells. These results indicate that PCU-SS could possess good subcutaneous histocompatibility and inhibitory effect on inflammatory cells in the vascular environment.

### Implantation of the Abdominal Aorta and Iliac Artery

**FIGURE 9:**
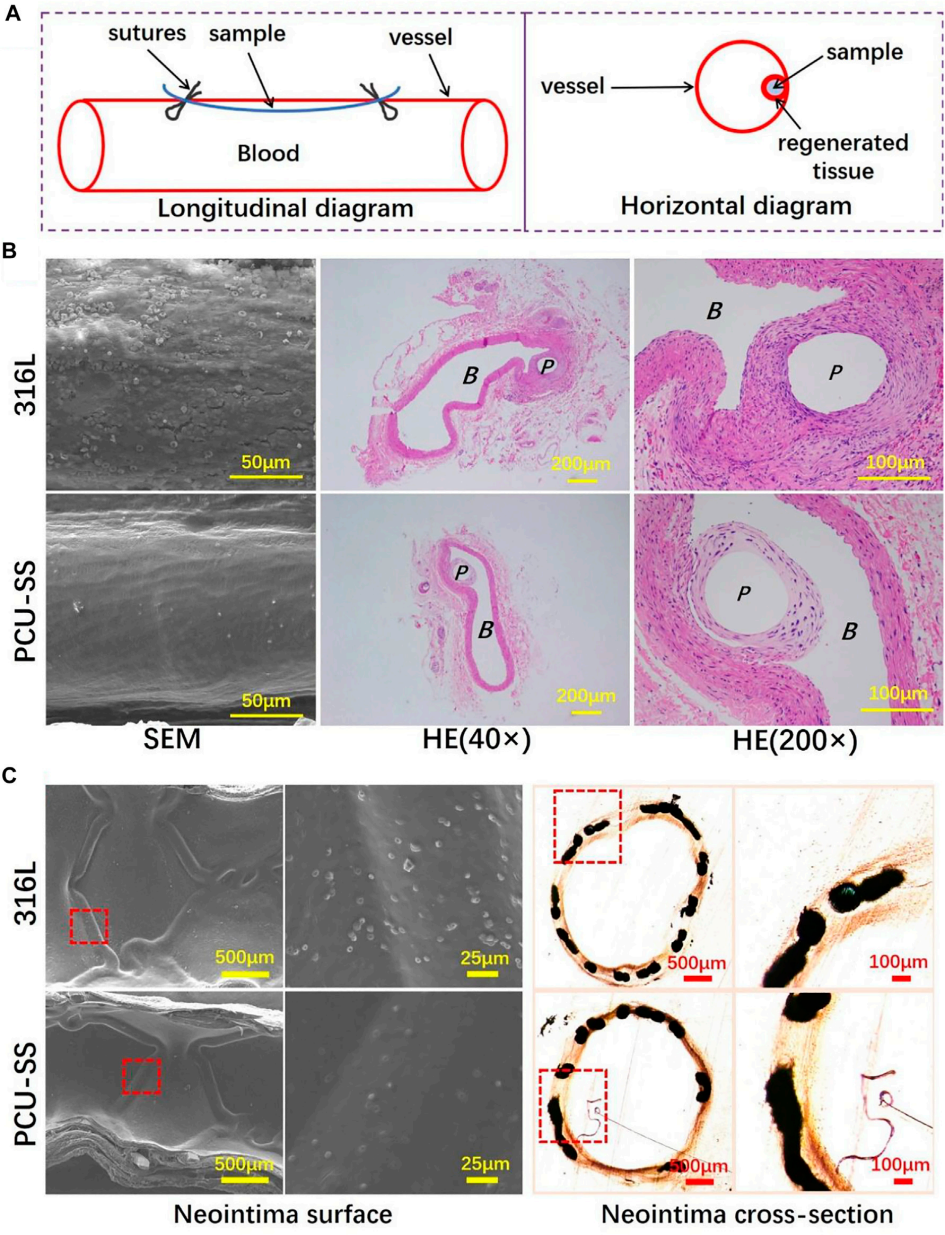
316L and PCU-SS samples are implanted into the abdominal aorta and iliac artery for 30 days **(A)** Longitudinal diagram of vascular implantation and horizontal diagram of the regenerated tissue around samples **(B)** SEM images of the regenerated tissue surface and HE staining results of paraffin sections of the regenerated tissue (P and B represent the location of sample implantation and blood flow, respectively) **(C)** The SEM results of the neointima surface and the histological images of neointima cross section after 30 days of iliac artery implantation.

To explore the biological behaviors in the vascular environment, the 316L wires before and after modification are implanted into the abdominal aorta of SD rats (Li et al., 2017; Wang et al., 2021). Due to the adhesion, proliferation, and migration of vascular cells, the implant surface will be covered by the neovascularization tissue after implantation. The longitudinal diagram of sample implantation and the transverse diagram of regenerated tissue around samples are shown in Figure 9A. As can be seen from the SEM results of sample surface tissues (Figure 9B), the regenerative vascular cells of 316L are not evenly arranged with a large number of platelets and red blood cell adhesion. By contrary, the vascular cells are uniformly covered on the PCU-SS surface with almost no adhesion of blood cells, indicating that the modified sample of PCU-SS could effectively promote the regeneration of healthy vascular tissues and inhibit the occurrence of thrombosis after implantation.

Subsequently, the regenerative tissues are stained with HE, and the results are shown in Figure 9B. The thickness of hyperplasia around PCU-SS (43.8 ± 11.9 μm) is significantly lower than that of 316L (57.2 ± 15.9 μm). The results are consistent with the evaluation results of SMC *in vitro*, which indicates that the modification of 316L with PCU-SS could effectively slow down the occurrence of hyperplasia. Moreover, immunofluorescence is used to further analyze the regenerated tissues. The expression level of CD 206 of PCU-SS is significantly higher than that of 316L, indicating a stronger anti-inflammatory ability (see Supplementary Figure S3A in Supplementary Material). In addition, the expression level of α-SMA is lower in 316L, while the expression level of OPN is higher, and PCU-SS showed an opposite trend to 316L (see Supplementary Figure S3B in Supplementary Material). This phenomenon indicates that PCU-SS can effectively promote the expression of contractile SMCs and inhibit the expression of synthetic SMCs.

Based on the research of implantation of abdominal aortas, endovascular stent implantation is used to further explore the biological behavior in the blood environment (Li et al., 2017; Wang et al., 2021). As a mature interventional medical device, vascular stents need multiple functions such as rapid endothelialization, anti-proliferation, and anti-coagulation (O’Brien and Carroll, 2009). The PCU-SS coating designed in this study contains GPx-like function, which can effectively mobilize the NO donor to decompose and then release NO in the body. After the scheduled time of implantation, the vessels containing stents are collected for subsequent analysis. As shown by SEM results in Figure 9C, both groups of samples are completely covered by neointima, but there are differences in blood cell adhesion. Visible blood cells and other blood components are discovered on the 316L surface, whereas adherent blood components on the PCU-SS surface are significantly reduced. The reason for this result is presumed to be that PCU-SS can continuously catalyze the release of NO from endogenous donors, thus improving the anticoagulant ability of PCU-SS. In addition to hemocompatibility, the hyperplasia thickness of neointima is also related to the long-term effectiveness of the stents. After tissue fixation, hard tissue sections are performed for microscopic observation and recording. Both bare metal stents (316L) and modified stents (PCU-SS) generate varying degrees of intimal hyperplasia. By contrast, PCU-SS has a stronger inhibitory effect on intimal hyperplasia. Unique NO catalytic release function would inhibit the adhesion and proliferation of SMCs and then slow down the intimal hyperplasia. Overall, corresponding to the result of *in vitro* biological evaluation and *in vivo* rat implantation, the result of stent implantation in this study illustrates the feasibility and effectiveness of the modified strategy.

## Conclusion

In this study, sulfur-mediated polycarbonate polyurethane, i.e., PCU-SS, is developed to improve biocompatibility with the body’s own defense mechanism. The GPx-like function of PCU-SS coating could induce NO release in the blood environment, hence improving the biocompatibility of the modified samples. The results of platelet adhesion *in vitro* and blood circulation *ex vivo* show the efficient capability of anti-thrombogenesis. Meanwhile, *in vitro* cell culture results confirm that PCU-SS has a good compatibility with EC and possesses the inhibitory effect on SMCs and MA. Moreover, the *in vivo* animal evaluations further demonstrate that the abilities of anti-inflammation, anti-thrombus, re-endothelialization, and anti-hyperplasia are markedly enhanced by PCU-SS coating. In summary, the modified strategy in this study has potential in the application of surface modification of blood-contacting devices, which could be a promising strategy for biocompatibility improvement of cardiovascular stents.

## Data Availability

The original contributions presented in the study are included in the article/Supplementary Material; further inquiries can be directed to the corresponding authors.
