# Supplementary material for: Sulfur-Mediated Polycarbonate Polyurethane for Potential Application of Blood-Contacting Materials
Source: Front Bioeng Biotechnol. 2022 Mar 9;10:874419. doi: 10.3389/fbioe.2022.874419 (PMC8959617; doi:10.3389/fbioe.2022.874419)
Supplement: Supplementary file 1 [file DataSheet1.docx]

Supplementary Material

Sulfur-mediated Polycarbonate-polyurethane for Potential Application of Blood-contacting Materials

Peichuang Li^a,b^, Wanhao Cai^b,c^, Xin Li^b,d^, Hong Zhang^a^, Yuancong Zhao^b,^*, Jin Wang^b,^*

^a^ Heze Branch, Qilu University of Technology (Shandong Academy of Sciences), Biological Engineering Technology Innovation Center of Shandong Province, Heze 274000, China

^b^ Key Laboratory of Advanced Technologies of Materials, Ministry of Education, School of Materials Science and Engineering, Southwest Jiaotong University, 610031, Chengdu, China

^c^ Institute of Physical Chemistry, University of Freiburg, Albertstraße 21a, Freiburg 79104, Germany

^d^ Department of Cardiology, Third People's Hospital of Chengdu Affiliated to Southwest Jiaotong University, Chengdu, 610031, China

^∗^ Corresponding authors.

E-mail addresses: zhaoyc7320@163.com (Y. Zhao); wangjin@swjtu.edu.cn (J. Wang)


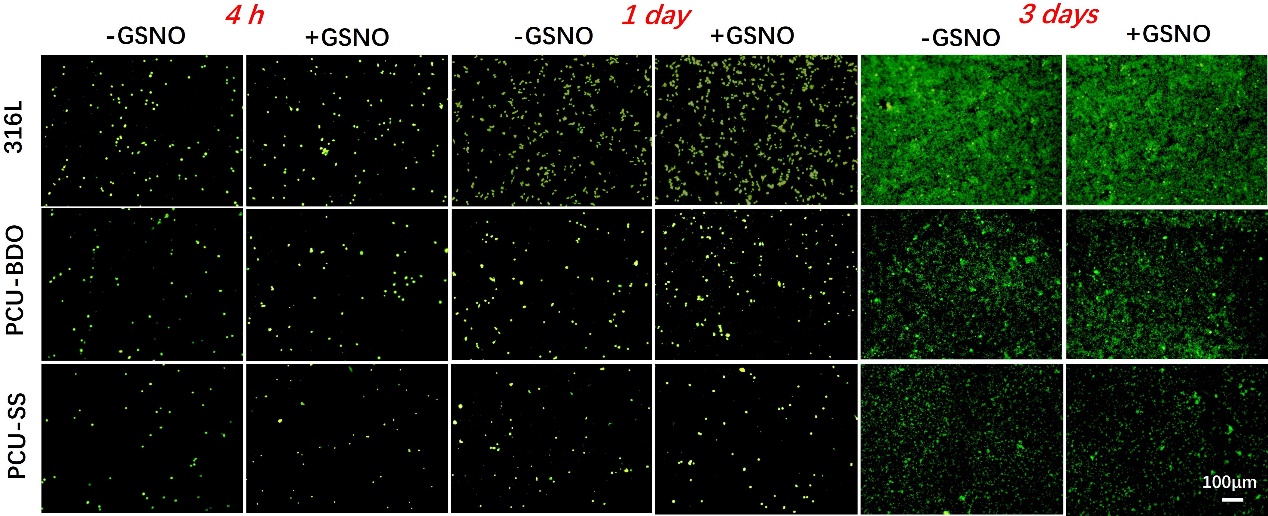


**Fig. S1.** Cal-AM fluorescence staining of MA on the different samples surface.


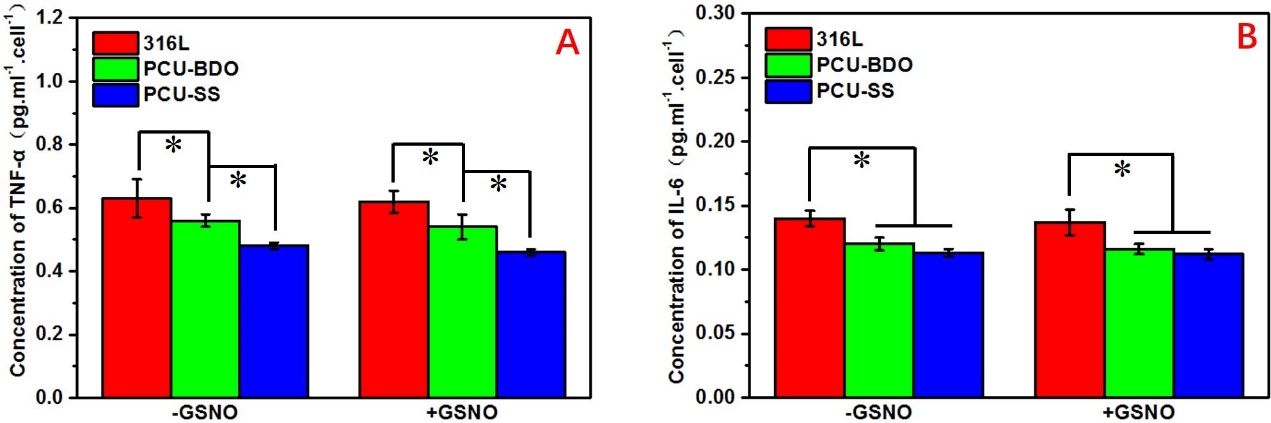


**Fig. S2.** (A) Quantitative analysis of TNF-α and (B) IL-6 of MA cultured for 3 days.


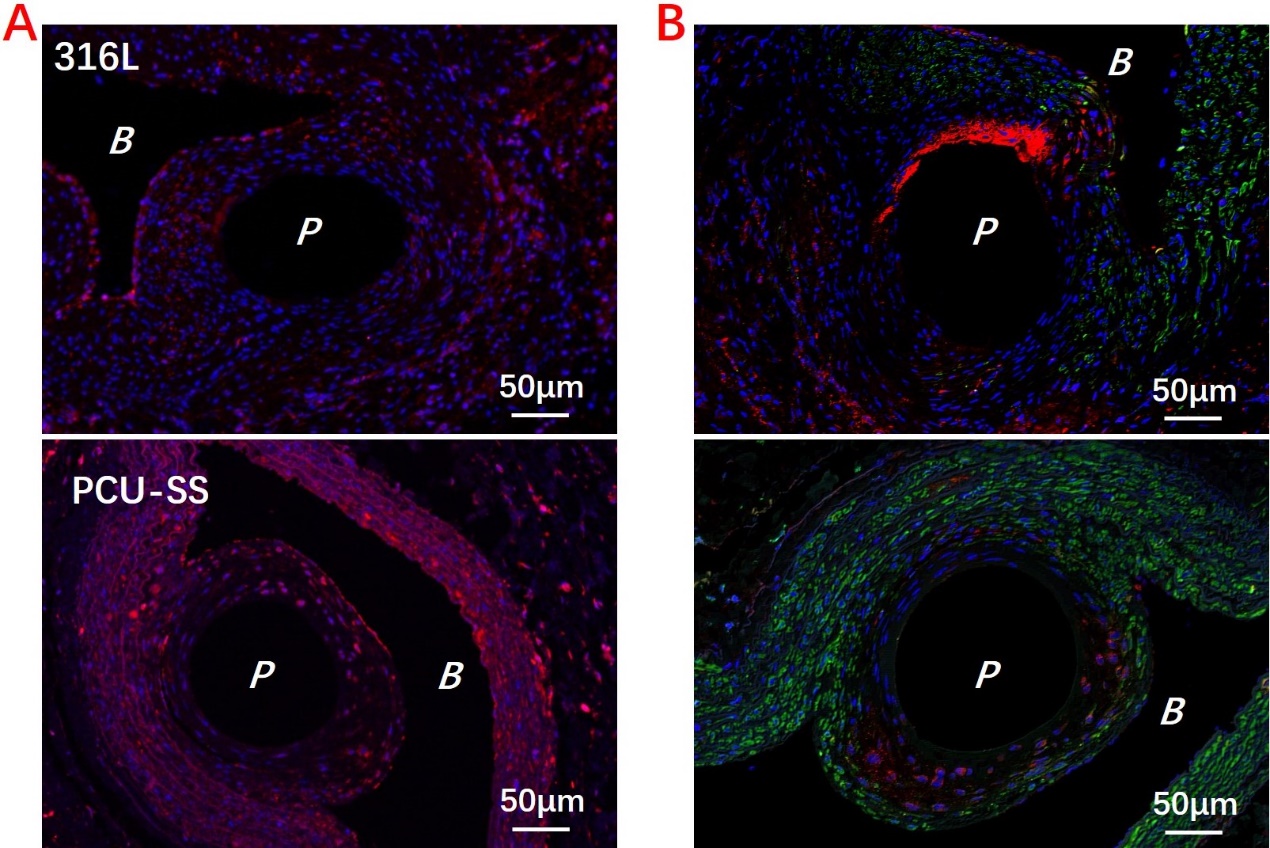


**Fig. S3.** Immunofluorescence staining results. (A) CD 206 staining of regenerated tissue (red is the positive expression of M2-type macrophage). (B) The results of α-SMA and OPN double staining of regenerated tissue (red is the positive expression of OPN of synthetic SMC, green is the positive expression of α-SMA of contractile SMC, *P* is the location of sample implantation, *B* is the location of blood flow).
